# Bilateral Semicircular Canal Dehiscence Analyzed Using the International Classification of Functioning, Disability, and Health (ICF) Framework: Case Report

**DOI:** 10.1002/ccr3.9687

**Published:** 2024-12-29

**Authors:** Ignacio Novoa‐Cornejo, Vijaya Prakash Krishnan Muthaiah, Victor Mercado‐Martinez, Gustavo Ulloa‐Alvarado, Carlos Pino‐Urrutia, Krishnamoorthy Gunasekaran

**Affiliations:** ^1^ Department of Rehabilitation Sciences, School of Public Health and Health Professions State University of New York at Buffalo Buffalo New York USA; ^2^ Instituto de Neurorrehabilitación y Balance Valparaíso Chile; ^3^ Exercise and Rehabilitation Sciences Institute, School of Physical Therapy, Faculty of Rehabilitation Sciences Universidad Andres Bello Santiago Chile; ^4^ Department of Medical Biochemistry, College of Health Sciences Dambi Dollo University Dambi Dollo Ethiopia

**Keywords:** audiological disorder, bilateral superior semicircular canal dehiscence, ICF framework, vestibular disorder

## Abstract

A severe case of bilateral superior semicircular dehiscence was presented in Instituto de Neurorrehabilitación y Balance, Chile. The patient reports hearing and vestibular problems in certain situations; a complete analysis is carried out from the clinical history to neurological laboratory studies and imaging to diagnose bilateral semicircular canal dehiscence finally. Health condition management is under the ICF model, which will allow for determining and classifying the problems and possible interventions for this interesting clinical case.

**Summary**

- The ICF framework is required for various otoneurological disorders, as demonstrated in this bilateral superior semicircular canal dehiscence case.
- It facilitates comprehensive assessment, guides clinical decisions, and enables dynamic symptom monitoring.
- Clinicians are encouraged to adopt this approach to enhance understanding of ICF in otoneurology.

## 1 Introduction

Bilateral semicircular canal dehiscence (B‐SCD) is a unique condition characterized by abnormal openings or thinning of the bony structures in the inner ear's semicircular canals [1]. It can manifest with various auditory and vestibular symptoms, significantly impacting an individual's overall well‐being. This case report focuses on exploring a specific case of bilateral SCD. Understanding the implications of B‐SCD on different aspects of individuals' lives, including auditory function, balance, and overall participation, is crucial. The International Classification of Functioning, Disability, and Health (ICF) framework is employed, allowing for a comprehensive evaluation of functioning status while considering contextual and personal factors [2, 3].

Through examining vestibular outcomes, including vestibular tests and assessments, valuable insights can be gained into the impact of B‐SCD on balance and spatial orientation. Computed tomography (CT) findings further aid in understanding the anatomical abnormalities within the semicircular canals, contributing to the underlying pathology of the condition [4]. The audiometric analysis is crucial in evaluating auditory function and associated symptoms in individuals with B‐SCD. By considering audiometric test results and related measurements, the impact of this condition on hearing abilities and communication skills can be assessed [1].

Incorporating the ICF framework into the analysis of B‐SCD enables a comprehensive evaluation of functioning implications across various domains, offering a holistic understanding of the condition. This approach contributes to developing tailored management strategies to address the specific needs of individuals with B‐SCD [2, 3]. This case report aims to explore the vestibular outcomes, CT findings, and audiometric analysis of individuals with B‐SCD. By utilizing the ICF framework, valuable insights into the functioning implications of the condition can be gained. This knowledge is a foundation for developing effective management strategies tailored to the needs of individuals with otoneurologic diseases.

## 2 Case Presentation

A 41‐year‐old male patient with no history of otalgia, ear overgrowth, hearing loss, or contusion. The patient sought consultation due to auditory discomfort, experiencing bilateral hyperacusis (increased sensitivity to sound), dizziness, and vertigo when pronouncing certain phonemes, including instances when water from the shower falls on the head.

## 3 Methods

A neurotologist specialist examination revealed no abnormalities. The auditory evaluation confirmed bilateral conductive hearing loss, particularly affecting the hearing thresholds between 125 and 1000 Hz frequencies, with ascending curves. Also, the bone conduction study showed thresholds below 0 dB at 250, 500, and 1000 Hz. Video Oculo Nystagmography (VNG) was conducted, including analysis of spontaneous nystagmus with and without ocular fixation and assessment of eye movements such as slow tracking and saccades. The results were within normal limits. Additionally, while, video head impulse test (v‐Hit) yielded normal findings, Cervical vestibular myogenic evoked potentials (c‐VEMPs) demonstrated a significant decrease in presentation thresholds.

**FIGURE 1:**
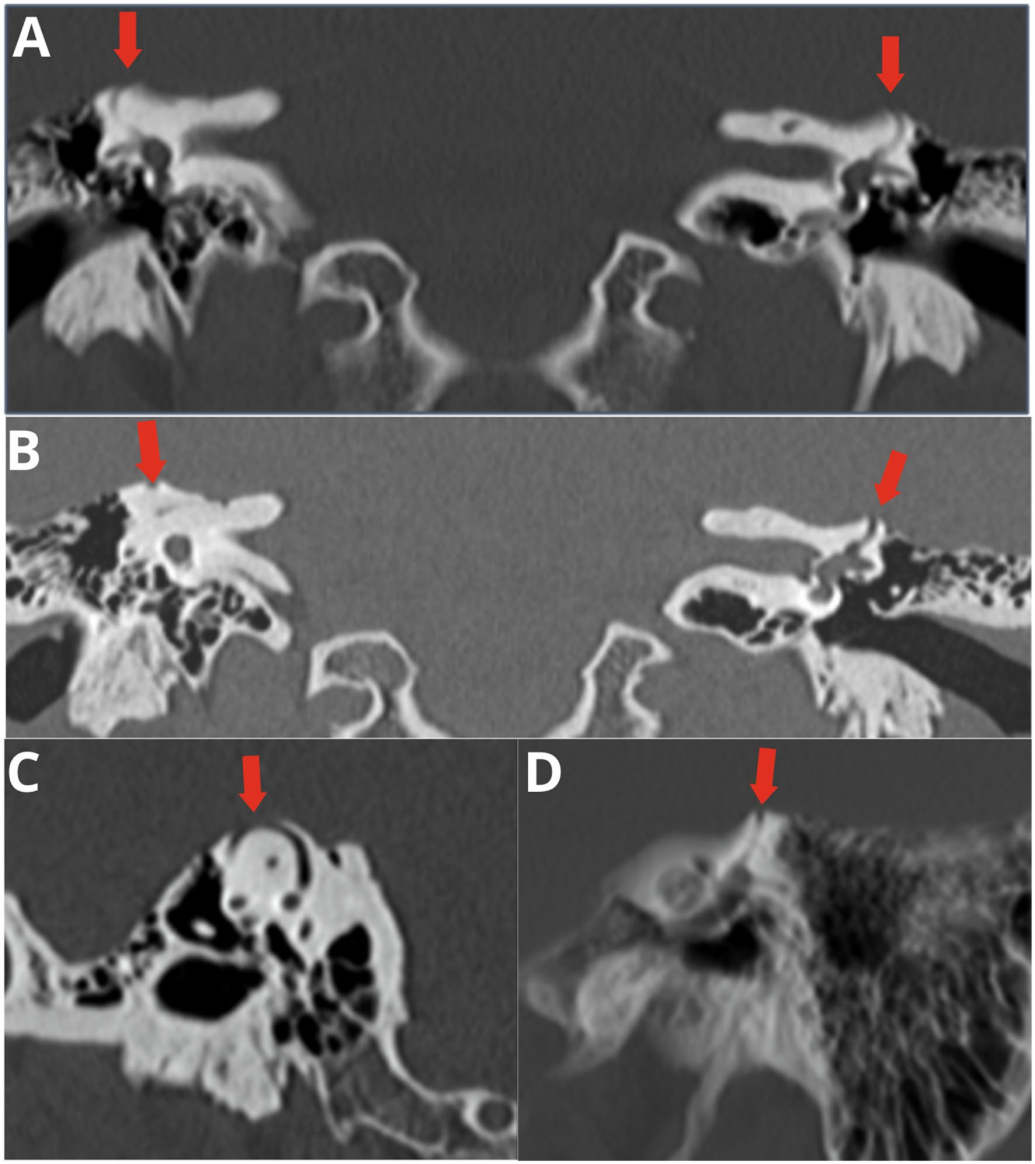
(A) Coronal reconstruction of petrous portions of temporal bones shows the absence of ossification of the bony roofs of the anterior semicircular canals (red arrows); (B) coronal oblique MPR and MIP reconstruction of petrous segments of temporal bones encompassing the uppermost segments of the anterior semicircular canals, showing the absence of a bony roof in both (red arrows); (C) oblique MPR reconstruction of the right anterior semicircular canal with dehiscence; (D) coronal oblique MPR, combined with MIP reconstruction of the petrous portion of the left temporal bone, demonstrates the lack of bony coverage of the cephalic edge of the anterior semicircular canal. MIP, maximum intensity projection; MPR, multiplanar reconstruction.

Furthermore, spontaneous nystagmus without ocular fixation was observed during pressure stimuli using the Valsalva Maneuver and sound stimulation while pronouncing the phoneme “M.” In particular, the production of the mentioned phoneme triggered an intense horizontal rotational nystagmus to the left, defined as “fremitus nystagmus” [4]. Based on the findings, a diagnosis of bilateral superior semicircular canal dehiscence (B‐SCD) is proposed. A high‐resolution ear CT scan with cuts smaller than 0.5 mm is recommended to confirm the presence of the bone defect (Figure 1).

The diagnosis of B‐SCD relies on a combination of characteristic symptoms, audiometric testing, and radiographic findings. Ward et al. established consensus criteria that include air‐bone gaps on audiometry and visible dehiscence of the superior canal on high‐resolution CT scans. Additional vestibular symptoms may consist of trigged dizziness or nystagmus in response to loud noises, as noted in the case report. While conservative treatment is reasonable in milder cases, surgery aimed at resurfacing or plugging the dehiscent canal can relieve symptoms [4]. The described bilateral B‐SCD repair surgery improves conductive hearing loss, though vestibular disturbances may persist [3, 4, 5].

## 4 Discussion

B‐SCD is characterized by the absence of bone covering the superior semicircular canals, leading to various auditory and vestibular symptoms. Articles shed light on this syndrome. Pereira et al. (2020) [5, 6] reported a case of B‐SSCD with bilateral conductive hearing loss and subtle vestibular symptoms. Bi et al. (2017) [7] explored superior semicircular canal dehiscense (SSCD) syndrome in depth, discussing its diagnosis and treatment options. Mercado et al. (2016) [8] presented a case study of SSCD syndrome, emphasizing its clinical features. Lee et al. (2016) [9] discussed a case of bilateral SSCD with the Tullio phenomenon, where sound‐induced dizziness occurs, as is the case in this report.

**FIGURE 2:**
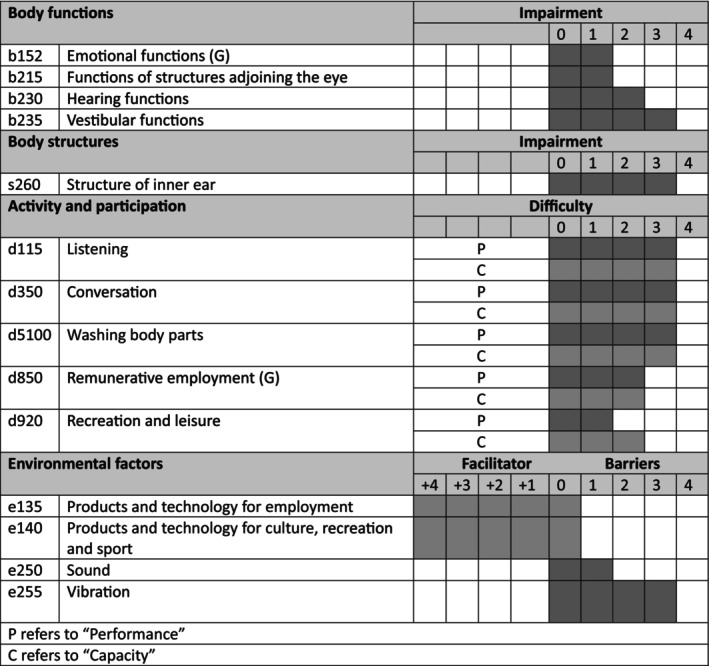
Functioning profile representation, the left column represents categories. The mild column represents the name of each categories, the numbers of the problem represent 0: no problem, 1: mild problem, 2: moderate problem, 3: severe problem, 4: complete problem, 8: not specified, 9: not applicable, In environmental factors, +4 represent complete facilitator, 0: nor barrier/facilitator until 4 that represents a complete barrier.

We developed an ICF assessment sheet to analyze this case. Using different sources of information like anamnesis interview, WHODAS 2.0, videonystagmography, v‐Hit, c‐VEMP, and CT. We created a functioning profile (Figure 2) where we selected specific categories according to patient necessities. The areas analyzed were body functions, structures, activity and participation, and environmental factors.

In the first domain, body functions, the patient manifests most impairment in hearing (b230) and vestibular function (b235), moderate and severe, respectively. We worked with the inner ear structure (s260) for body structure; the classification was severe, the nature is “aberrant dimension,” and the location is 3, meaning “both.”

The activity and participation were analyzed according to patient requirements; listening (d115), conversation (d350), and washing body parts (d5100) were categories that the patient claimed like relevant in his life and about the qualification are severe limitation in functioning profile. According to the interview, remunerative employment (d850) and recreation and leisure (d920) are mild restriction. In the environmental factors, we described his facilitators as products and technology for employment and precuts and technology for culture, recreation, and sport, e135 and e140, respectively. Nevertheless, we can describe barriers, sound (e250), and vibration (e225), two variables essentials to consider; the first one has a mild impairment, and the second is severe.

The use of the International Classification of Functioning, Disability, and Health (ICF) in the field of otoneurology can be a great help in the evaluation and clinical reasoning for diagnosis and treatment [10, 11, 12, 13]. As previously mentioned, the patient expressed that auditory difficulties were the most challenging, affecting their performance and daily activities. This is supported by evidence suggesting that patients with SSCD often experience auditory difficulties [6, 7, 8, 14, 15]. Although B‐SCD is atypical, its structural characteristics are described [16]. As a structural condition, its solution necessarily requires a structural approach. This is where the functioning profile of the ICF helped guide this clinical case. The intervention focused on performance education in daily variables such as personal hygiene, adapting work methods, providing education about the patient's health condition, and avoiding triggering factors. Strategies were developed to mitigate exposure to sound and vibration, which were identified as crucial environmental barriers. Regular follow‐ups using the ICF functioning profile enabled dynamic management adjustments. An interdisciplinary approach involving ENT surgeons, imaging specialists, and neurologists ensured comprehensive care. The patient was educated about surgical options and the importance of ongoing monitoring, particularly considering future interventions like cochlear implantation. This ICF‐guided approach addressed the multifaceted impacts of B‐SCD while providing a framework for tailored, ongoing management, emphasizing the importance of monitoring the dehiscence and its symptoms. Bilateral superior semicircular canal dehiscence is an unusual condition with multiple implications regarding the patient's quality of life. Managing such complex cases requires an interdisciplinary team of ENT surgeons, imaging specialists, neurologists, and neurosurgeons. Usually, this pathology is discovered at a younger age. However, due to the lack of access to medical treatment, this pathology must also be considered in the differential diagnosis in older cases; therefore, this evaluation method is recommended for healthcare teams to guide intervention decisions best for patients.

## 5 Conclusion

Utilizing the ICF framework revealed auditory limitations to be the predominant disability, negatively affecting daily functioning, conversations, and work. While structural aberrancy underlies the root pathology in B‐SCD, a functioning‐based management plan focused on performance education tailored to the patient's activity priorities proved helpful. Although surgery may be indicated, conservative management with avoidance of sound and vibration triggers and monitoring symptom progression using the ICF functioning profile is a reasonable initial approach, as exemplified in this case.

## Author Contributions

**Ignacio Novoa‐Cornejo:** investigation, methodology, writing – original draft. **Vijaya Prakash Krishnan Muthaiah:** formal analysis, validation, writing – review and editing. **Victor Mercado‐Martinez:** methodology, writing – original draft. **Gustavo Ulloa‐Alvarado:** investigation, methodology. **Carlos Pino‐Urrutia:** formal analysis, investigation, methodology. **Krishnamoorthy Gunasekaran:** formal analysis, validation, writing – review and editing.

## Consent

Written informed consent was obtained from the patient to publish this report following the journal's patient consent policy.

## Conflicts of Interest

The authors declare no conflicts of interest.

## Data Availability

The clinical identified data is available on reasonable request.
